# Meetings of Societies

**Published:** 1907-06

**Authors:** 


### MEETINGS OF SOCIETIES.

### Bristol Medico-Chirurgical Society.

*March 13th*, 1907.

Mr. James Taylor, President, in the Chair.

The meeting was devoted to the consideration of **Cases and Specimens** illustrating various phases of **Syphilis.**

Professor Walker Hall and Dr. Carey Coombs demonstrated **Pathological Specimens,** macro- and microscopical.

Dr. Michell Clarke showed sections illustrating **Syphilitic Diseases of the Brain and Spinal Cord.**

The following cases were shown :—**Charcot's Disease of the Knee Joints,** by Dr. Alexander ; **Disseminated Choroiditis,** by Dr. Ogilvy ; **Relapsing Chancre, Spontaneous Fracture of Tibia,** and **Tertiary Periostitis of Radius,** by Mr. Hey Groves ; **Secondary Syphilis** with **Sloughing Tonsil** and **Suppurating Glands in the Neck, Necrosis of the Lower Jaw, Congenital Ulceration of Nares and Palate,** and **Recurrent Gummatous Ulceration of the Scalp,** by Dr. Kenneth Wills.

Patient treated with **Intra-muscular Injections of Lactate of Mercury**, and a patient with a **Tubercular Syphilide,** the disease having been acquired innocently, by Dr. Nixon.

---

*April 10th*, 1907.

Mr. James Taylor, President, in the Chair.

Mr. C. A. Morton showed a specimen of **Large Ovarian Tumour,** weighing 10½ lb. and measuring 13 in. by 10 in. by 6 in., which had been removed from a child of ten years of age. Sections showed the tumour was an adeno-sarcoma.

Dr. E. C. Williams showed (1) A boy with **Enlarged Liver and Spleen.** The spleen, first noticed about Christmas last, extended downwards nearly to the iliac crest and forwards to the middle line. The liver edge could be felt two inches below the costal margin. There was slight jaundice, slight clubbing of the fingers and toes. There was a faint systolic murmur at the heart's apex. Leucocytosis was distinct, pointing to the presence of an infective agent. The disease did not appear to be due to any of the exanthemata. (2) A case of **Sporadic Cretinism** in a girl aged 3½ years, after six months' treatment with thyroid entract. There had been very marked improvement. Nothing abnormal had been noticed in the child until after whooping-cough at four months of age.

Dr. EDGEWORTH read notes on a case of **Double Consciousness.** In the dreams of sleep there is no sharp division between perceptions and conceptions, for the latter are projected and have the character of perceptions; secondly, in dreams, there is an entire absence of logical control; and thirdly, the emotional side of mental processes is exaggerated. The condition of hypnosis is closely related to that of dream-consciousness. Some of the hypnotised remember the events of the hypnotic state; others entirely forget them on waking. In spontaneous somnambulism the individual possesses sense-perceptions and translates his motor ideas into acts. The condition of double personality is clearly akin to somnambulism, but differs from it in that its onset is in waking life, and also in its prolonged duration. The example the speaker described was that of a man who found himself, he thought, in Old Market Street, Bristol, and asked his way to the police station, but could not tell his name nor where he came from, nor how he happened to be in Bristol. He was brought to the Royal Infirmary. A clue was found through his shirt markings, and it was discovered that a man had been missing from the town so discovered. A police officer, armed with this information, asked the man if he was Mr. —— of a certain town. The man said yes, with astonishment. In a little while the man's memory returned, but he could not recall leaving his native town, nor any event since then until coming partly to himself in Old Market Street. He had been missing from home a month, but what he had done in that month remains a mystery. There are many similar cases on record. In one, the man when hypnotised recollected what he had done during six weeks of aberration, but could not recollect it unless hypnotised. It is the subconscious or infra-liminal ideas, thoughts, sensations and perceptions which form the basis of dream-consciousness, somnambulism, hypnosis and double personality.

Dr. WALTER SWAYNE read a paper on **Cerebral Complications in Pregnancy and Parturition.**

Dr. CAREY COOMBS read a paper on **Rheumatic Carditis in Childhood.**

Dr. BERTRAM ROGERS read notes on a case of **Sclerodermia with Trophic Lesions.**

J. LACY FIRTH.

H. F. MOLE, *Hon. Sec.*

